# A comparison of patient-reported quality between inpatient services for mental and physical health: A tertiary-hospital-based survey in China

**DOI:** 10.3389/fpsyt.2023.1090892

**Published:** 2023-02-09

**Authors:** Wei Zhou, Shuiyuan Xiao, Guanqing Xie, Feiyun Ouyang, Bihua Luo

**Affiliations:** ^1^Research Center for Public Health and Social Security, School of Public Administration, Hunan University, Changsha, Hunan, China; ^2^Xiangya School of Public Health, Central South University, Changsha, Hunan, China; ^3^Clinical Nursing Teaching and Research Section, The Second Xiangya Hospital, Central South University, Changsha, Hunan, China; ^4^National Clinical Research Center for Metabolic Diseases, Department of Metabolism and Endocrinology, The Second Xiangya Hospital, Central South University, Changsha, Hunan, China

**Keywords:** mental health services, physical health services, patient-reported quality, inter-sectoral comparison, inpatient services, China

## Abstract

**Background:**

It is widely acknowledged that quality of mental health services is routinely worse than physical health services across countries. However, studies separately investigating mental health services often report high-level satisfaction, even comparing with physical health services. Therefore, this study aimed to compare patient-reported quality between inpatient services for mental and physical health in China.

**Methods:**

An inpatient survey was conducted among service users of mental and physical health services. Patient-reported quality was measured by the responsiveness performance questionnaire after patient discharge and based on patients' multiple experiences of hospitalization in the past 3 years. Chi-square tests were performed to compare the two patient groups' ratings on inpatient services for mental and physical health, and multivariate logistic regression was performed to adjust covariates in the group comparison.

**Results:**

Inpatient services for mental health were rated better than those for physical health on “treating with respect” (AOR = 3.083, 95% CI = 1.102–8.629) and “choosing a healthcare provider” (AOR = 2.441, 95% CI = 1.263–4.717). However, mental health services had poorer ratings on “asking patient's opinions” (AOR = 0.485, 95% CI = 0.259–0.910). For other responsiveness items, no significant difference was detected between the two types of inpatient services.

**Conclusion:**

Mental health inpatient services provided by China's tertiary hospitals could perform as well as physical health inpatient services in most aspects and even better perform regarding dignity and choice of healthcare providers. However, neglecting patients' voices is more severe in inpatient services for mental health.

## Introduction

Due to lower priority, insufficient funding and inadequate human resources for mental health, the quality of mental health services is routinely worse than that of physical health services across countries (1–4). Many patients with severe mental disorders sometimes experience even poorer care associated with abuses of their human rights (1, 2).

However, studies on mental health services often report a high level of patient satisfaction (5, 6), even in comparison with that in studies on physical health services. For example, a survey conducted in 57 hospitals in five European countries reported that the mean score of patient satisfaction with psychiatric inpatient care was 7.29 ± 2.16 out of 10 (7). A systematic review on psychiatric inpatient services also found good patient satisfaction in most studies, with a mean satisfaction score of 23.53 out of 32 or a satisfaction proportion range between 60% and 68% (8). In contrast, a multi-country survey involving 488 hospitals in eight European countries and 617 hospitals in the United States showed that high patient satisfaction ratings for physical health services ranged only from 35% to 61% (9).

In China, mental health services also suffer from insufficient resources and inequitable distribution of resources (10, 11). Before the release of the National Mental Health Law in 2012, the psychiatric institutions in China were even criticized by the international community for right violations of psychiatric patients (12, 13). Even though, a national survey among inpatients from 32 psychiatric hospitals reported a mean satisfaction score of 23.3 ± 2.4 out of 25 (6). In comparison, another national survey on patient satisfaction of physical health services reported an overall score of 7.61 ± 2.12 out of 10 for inpatient care (14).

Due to differences in study designs, care settings, assessment instruments and scoring methods, comparison based on the above studies can hardly provide a reliable conclusion to questions of whether and how much quality gap exists between services for mental and physical health. Therefore, we designed an inpatient survey to compare patient-reported quality between services for mental and physical health in the context of China. The inter-sectoral comparison could better detect problems existing in mental health services and inform quality improvement accordingly.

## Methods

### Study design and setting

A cross-sectional survey was designed under the recommended checklist of STROBE (Strengthening the Reporting of Observational Studies in Epidemiology) for cross-sectional studies, including study design, setting, participants, variables and measurement and study size (15).

The survey was conducted from 1st June and 31st July, 2018 among service users of mental and physical health services, who were consecutively recruited from the inpatient wards of the Psychiatric Department (PD) and the Endocrinology Department (ED) in one of the largest public tertiary general hospitals in Changsha, the capital city of Hunan Province, China. The PD had 150 mental health professionals and 6 inpatient wards with 231 beds, providing services for approximately 130,000 outpatients and 4,200 inpatients per year for surrounding cities and rural areas. The ED had 121 medical professionals and 3 inpatient wards with 126 beds, with patient volume of over 110,000 (outpatient) and 4,000 (inpatient) person times per year. The ED was chosen for comparison for two reasons: (1) the majority of patients in the ED were diagnosed with chronic physical illnesses, such as diabetes mellitus and metabolic disorder. Like patients from the PD, they also had multiple encounters with health care throughout their treatment lasting for years. (2) Apart from the chronic nature of patients' medical conditions, both the ED and PD were ranked as the top clinical departments in the selected hospital, which were also comparable at the sector level.

### Participants

Eligible patients were 18 years old or above; mentally and physically capable of completing interviews according to their clinical records; discharged between 1st June and 31st July in 2018. Patients were excluded, if they were: (1) admitted to the PD for substance abuse only; (2) unwilling to receive a post-discharge telephone survey.

An a priori sample size calculation was determined according to the formula for comparing two population proportions: *n* = $\frac{{\left(Z_{\alpha /2}+Z_{\beta }\right)}^{2}\left[p_{1}\left(1-p_{1}\right)+p_{2}\left(1-p_{2}\right)\right]}{{\left(p_{1}-p_{2}\right)}^{2}}$. Here α was set as 0.05 at a confidence level of 95%, and β was set as 0.2 for a power of 80%. Based on previous studies (8, 9), the expected satisfaction proportions mental health services (*p*_1_) and physical health services (*p*_2_) were set as 65% and 48% respectively. For the comparison, we calculated a minimal sample size of 130 users for each type of services.

### Study variables and measures

#### Patient-reported quality of inpatient services

Patient-reported quality, as the dependent variable, was measured by the concept of responsiveness proposed by the World Health Organization (WHO) (16). As an alternative to patient satisfaction, responsiveness only focuses on a healthcare system's performance of non-medical aspects (17). As responsiveness could describe healthcare quality apart from positive health outcomes and non-impoverishment (18), it was particularly appropriate for quality assessment across sectors and types of diagnosis (17, 19). The responsiveness performance questionnaire, the operationalized instrument for measuring responsiveness, had been widely validated and used in evaluations on physical and mental health services (17, 18, 20–23). The 15-item questionnaire assesses eight domains: dignity, confidentiality, communication, autonomy, choice, social support, quality of basic amenities, prompt attention (22). Each item was rated on a five-point scale, with higher ratings indicate better responsiveness performance. To maximally cover multiple service providers, participants from the PD and ED were asked to rate based on their inpatient experiences of mental and physical health services in the past 3 years, rather than their last experiences in the survey hospital.

#### Covariates

(1) Social-demographic and clinical characteristics: A self-designed questionnaire was used to collect each participant's age, gender, marital status, education, employment, diagnosis, treatment years and hospitalization experiences in different hospitals, which were frequently tested and reported as determinants of patient-reported healthcare quality in previous studies (6, 8, 24).
(2) Reporting behaviors on healthcare quality: As self-reported measures were prone to personal biases in reporting styles, participants' reporting behaviors on responsiveness were measured by vignettes (25). Eight vignettes were selected from the World Health Survey and the WHO Study on Global Ageing and Adult Health (SAGE) (2007–2008). The selection, translation and adoption process and full texts of the eight vignettes used in this study was reported elsewhere (26). In brief, each vignette provided one scenario of people's experiences with health services to test one responsiveness domain that corresponded to the responsiveness performance questionnaire. Respondents' ratings on the vignettes served as a judging benchmark of their responsiveness ratings on experiences. In the present study, significantly different reporting behaviors were detected between patients from the PD and ED on several responsiveness performance items (Appendix 1 in Supplementary material).

### Data collection

The study was approved by the Institutional Review Board of the Xiangya School of Public Health, Central South University (XYGW-2018-01). Potentially eligible patients were approached at bedside on the day or 1 day before patient discharge. After explaining the study purpose and procedures, written informed consent was obtained from all participants before data collection.

To minimize the influences of hospitalization status on patients' ratings on responsiveness (27), the data collection process was divided into two stages: (1) a face-to-face survey on the day or 1 day before patient discharge, which collected participants' social-demographic and clinical information and reporting behaviors on healthcare quality; (2) a telephone survey within 1 week after patient discharge, which collected patient-reported quality of inpatient services. Measures, including asking participants' call time preference and sending notifying text messages in advance, were used to increase the response rate in the second stage. Participant recruitment and both stages of surveys were conducted by postgraduates with a background in public health.

### Data analysis

Group differences of social-demographic and clinical characteristics were compared between patients from the PD and ED, by chi-square tests or Mann–Whitney U tests. In accordance with WHO's approach in the Multi Country Service Study, ratings of the responsiveness performance questionnaire were dichotomized into “good performance” (ratings ≥ 4) and “poor responsiveness performance” (ratings ≤ 3) (20). Chi-square tests were performed to identify rating differences of inpatient experiences between mental and physical health services. For each responsiveness performance item, a multivariate binary logistic regression model was performed to adjust covariates in the group comparison between the PD and ED. Adjusted odds ratio (AOR) and 95% confidence intervals (CI) were reported. No missing data were replaced. All analyses were performed in SPSS 23.0 and statistical significance level was set as 0.05.

## Results

### Participant characteristics

Overall, we approached 298 patients from PD and 211 patients from ED; 234 patients from the PD and 181 from the ED completed the face-to-face survey; 168 patients from the PD and 132 patients from the ED finished the telephone survey. In both departments, participants retained in the post-discharge telephone survey were younger and more likely to be employed (*P* < 0.05) (Appendix 2 in Supplementary material).

Comparative analyses (Table 1) between patients from the two departments yielded nonsignificant results in gender and employment status; however, patients from the PD were younger (*P* < 0.001), less likely to be married (*P* < 0.001) or treated in multiple hospitals (*P* < 0.001), had higher educational level (*P* < 0.001) and shorter years of treatment (*P* = 0.005). Among patients from the PD, 20.1% of them were diagnosed with schizophrenia-spectrum disorders, 46.9% with mood disorders, 18.9% with anxiety disorders. For patients from the ED, 65.7% of them were diagnosed with diabetes mellitus, 13.1% with metabolic bone disease, and 6.8% with hypofunction and other disorders of pituitary gland.

**Table 1:** Participant characteristics.

**Variables**	**Patients from the PD (*n* = 168)**	**Patients from the ED (*n* = 132)**	*P* ^*^
Age, mean, median (IQR)	35.3, 32 (23, 47)	53.3, 55 (43, 65)	<0.001
Male, *n* (%)	83 (49.4)	66 (50.0)	0.918
Marital status^a^, *n* (%)			<0.001
Married	90 (53.6)	102 (77.3)	
Unmarried	78 (46.4)	30 (22.7)	
Education, *n* (%)			<0.001
≤Primary school	11 (6.5)	42 (31.8)	
Junior high school	39 (23.2)	33 (25.0)	
Senior high school	50 (29.8)	28 (21.2)	
≥University	68 (40.5)	29 (22.0)	
Employment^b^, *n* (%)			0.293
Employed	69 (41.1)	46 (35.1)	
Unemployed	99 (58.9)	85 (64.9)	
Years of treatment, mean, median (IQR)	3.0, 1 (0, 5)	6.8, 2 (0, 11)	0.005
Hospitalization in multiple hospitals in past 3 years, *n* (%)	50 (29.9%)	66 (50.0%)	<0.001

IQR, interquartile range.

* P value by χ^2^ tests or Mann–Whitney U tests.

a Married includes married and cohabited, unmarried includes single, divorced, and widowed.

b Employed includes employed and self-employed, unemployed includes unemployed and retired.

### Comparison of patient-reported quality between mental and physical health services

Table 2 presents results of chi-square tests on responsiveness performance ratings on inpatient services for mental and physical health. Compared to the ED patients' ratings on physical health services, significantly fewer patients from the PD rated mental health services as “good performance” on the following items: (1) “information of alternative treatments or tests” (PD = 27.9%, ED = 41.1%; *P* = 0.017), from the domain of autonomy; (2) “asking users' opinions when making decisions” (PD = 42.7%, ED = 55.0%; *P* = 0.036), also from the domain of autonomy; (3) “family/friend visits during hospitalization” (PD = 90.8%, ED = 97.7%; *P* = 0.016), from the domain of social support. However, mental health services were more frequently rated as “good performance” than physical health services on “treating with respect” from the domain of dignity (PD = 94.0%, ED = 86.4%, *P* = 0.023) and on “choosing a healthcare provider” from the domain of choice (PD = 64.6%, ED = 51.6%, *P* = 0.026). For the other 10 items, no significant difference of patients' ratings was detected between mental and physical health services.

**Table 2:** Comparison of responsiveness performance ratings between mental health and physical health inpatient services.

**Domain/item**	**Mental health services^a^**	**Physical health services^a^**	**Chi-square/df**	***P***
	***n*** **(%)**	***n*** **(%)**		
Domain: Dignity				
Q1. Treating with respect	158 (94.0)	114 (86.4)	5.158/1	0.023
Q2. Physical privacy during examination and treatments	149 (89.8)	113 (86.3)	0.863/1	0.353
Domain: Confidentiality				
Q3. Talking privately, without overhearing	53 (31.9)	30 (22.9)	2.963/1	0.085
Q4. Keeping patient information confidential	148 (89.2)	111 (86.0)	0.655/1	0.418
Domain: Communication				
Q5. Communication understandable	139 (82.7)	114 (87.0)	1.038/1	0.308
Q6. Enough time for questioning	135 (80.4)	99 (76.2)	0.768/1	0.381
Domain: Autonomy				
Q7. Information of alternative treatments or tests	46 (27.9)	53 (41.1)	5.654/1	0.017
Q8. Asking patient's opinions when making decisions	70 (42.7)	71 (55.0)	4.416/1	0.036
Domain: Choice				
Q9. Choosing a healthcare provider	104 (64.6)	65 (51.6)	4.941/1	0.026
Domain: Social support				
Q10. Family/friend visits during hospitalization	148 (90.8)	125 (97.7)	5.812/1	0.016
Q11. Contacting with outside during hospitalization	150 (92.6)	125 (97.7)	3.738/1	0.053
Domain: Quality of basic amenities				
Q12. Cleanliness of inpatient wards	155 (93.4)	115 (89.8)	1.201/1	0.273
Q13. Overall comfortableness of inpatient wards	149 (89.8)	112 (87.5)	0.370/1	0.543
Domain: Prompt attention				
Q14. Traveling time to hospital	65 (39.6)	57 (45.6)	1.035/1	0.309
Q15. Waiting time of being admitted	99 (62.7)	73 (58.9)	0.419/1	0.518

a Rating as “good performance.”

After controlling cofounders from participants' social-demographic and clinical characteristics and reporting behaviors on healthcare quality, responsiveness performance ratings remained significantly different between the two types of services on three items (Table 3). Specifically, mental health services were more likely to be rated as “good performance” than physical health services on “treating with respect” from the domain of dignity (AOR = 3.083, 95% CI = 1.102–8.629, *P* = 0.032) and “choosing a healthcare provider” from the domain of choice (AOR = 2.441, 95% CI = 1.263–4.717, *P* = 0.008). However, “asking user's opinions when making decisions” from the domain of autonomy tended to have poorer performance in mental health services (AOR = 0.485, 95% CI = 0.259–0.910, *P* = 0.024).

**Table 3:** Multivariate logistic regression analysis to control confounders in quality comparison between mental health and physical health inpatient services.

**Domain/item**	**AOR (95% CI)**	***P***
Domain: Dignity		
Q1. Treating with respect	3.083 (1.102–8.629)	0.032
Q2. Physical privacy during examination and treatments	1.634 (0.593–4.505)	0.343
Domain: Confidentiality		
Q3. Talking privately, without overhearing	1.441 (0.704–2.950)	0.318
Q4. Keeping patient information confidential	1.311 (0.502–3.421)	0.580
Domain: Communication		
Q5. Communication understandable	0.873 (0.358–2.132)	0.766
Q6. Enough time for questioning	1.746 (0.801–3.807)	0.161
Domain: Autonomy		
Q7. Information of alternative treatments or tests	0.612 (0.325–1.151)	0.128
Q8. Asking patient's opinions when making decisions	0.485 (0.259–0.910)	0.024
Domain: Choice		
Q9. Choosing a healthcare provider	2.441 (1.263–4.717)	0.008
Domain: Social support		
Q10. Family/friend visits during hospitalization	0.192 (0.021–1.755)	0.144
Q11. Contacting with outside during hospitalization	0.208 (0.025–1.697)	0.143
Domain: Quality of basic amenities		
Q12. Cleanliness of inpatient wards	1.499 (0.505–4.443)	0.466
Q13. Overall comfortableness of inpatient wards	1.733 (0.686–4.382)	0.245
Domain: Prompt attention		
Q14. Traveling time to hospital	1.395 (0.739–2.636)	0.305
Q15. Waiting time of being admitted	1.425 (0.747–2.719)	0.282

Physical health services were set to be the reference group.

## Discussion

To our best knowledge, this study was the first attempt to compare quality and detect gaps between mental and physical health services based on patient assessment. In addition, we recruited inpatients from two clinically matched departments from the same hospital; surveys in the two departments were administered with the same instruments and under the same interview procedures. This tackles the problem of comparability across existing studies, which separately investigated patient satisfaction of mental or physical health services with different study designs, settings and assessment instruments (6–9, 14, 27, 28).

The participants reported that mental health services performed as well as physical health services on 12 responsiveness performance items of 5 domains (confidentiality, communication, social support, quality of basic amenities and prompt attention). This is different from the existing knowledge that the quality of mental health services is routinely worse than physical health services (1). The results of non-inferiority between the two types of services could be attributed to two potential explanations. Firstly, as the two clinical departments were selected from the same hospital, there were institutionally standardized training schemes for service providers' practices (like communication with patients), same regulations on hospital management (like visiting regulations and admission procedures) and similar basic amenities in inpatient wards. The institutional-level consistency could promote the consistency of patients' experiences. Secondly, the Chinese government has been committed to strengthening the mental health system since 2000 (29, 30). A series of national policies has been released to improve mental health services, which include the Guidelines for the Development of National Mental Health Care System (2008–2015) and the Plan for Construction and Development of Mental Health Care System (2010) (11, 31). Specifically to hospital-based services, the Proposal on Construction Mental Health Institutions (2008) and its special funds of the grant by the central government has greatly improved the infrastructure of mental health institutions across China (32); the National Mental Health Law (2012) has provided comprehensive regulations on treatment of mental disorders and rights protection of patients (33); the National Project of Specialty-Transfer Training to be Psychiatrists since 2015 has largely scaled up mental health services (34). In our study, as inpatient experience in multiple hospitals was included as a covariate in the multivariate regression analyses, it also indicated that the non-inferiority of patient assessment between mental and physical health services was on a larger scale beyond a single hospital.

Though 86.4% patients from the ED rated physical health services as “good performance” on “treating with respect” (Domain: Dignity), mental health services outperformed physical health services on this item both before and after covariate adjustment. On one hand, this could be regarded as a positive result of the implementation of the National Mental Health Law (2012), which advocates human dignity and right protection of patients with mental disorders (35). On the other hand, this might also be related to the difference of tools and methods for diagnosing and treating mental disorders and physical diseases. In general, services for physical health more rely on biochemical tests and techniques. In addition to biochemical techniques, symptom examination, counseling and psychotherapy are also very important clinical practices in psychiatry. The above clinical practices require professionals to better understand patients and create clinical relationships (36), which might provide extra comfort to patients' feelings and promote their feeling of be respected.

For the domain of choice, mental health services also had better performance than physical health services both before and after covariate adjustment. This indicates that patients with mental disorders could more often access to inpatient services provided by hospitals or clinicians they are happy with than their counterparts with chronic physical conditions. Without a strict referral system, patients in China could go directly to any hospital as they wish (37); therefore, hospital capacity and healthcare utilization of patients are key to the domain of choice. By the end of 2018, there were 569,031 and 1,639,053 hospital beds for psychiatry and internal medicine, respectively (38). The China National Health Services Survey of 2018 reported that the rates of hospital admission were 0.8‰ for mental and behavioral disorders and 5.8‰ for endocrine, nutritional and metabolic diseases (39). Despite fewer mental health resources, much lower utilization of care among patients with mental disorders in China (39–41) makes mental health services seemingly more available for choice than physical health services.

After covariate adjustment, “asking patient's opinions when making decisions” (Domian: Autonomy) was the only item with poorer patient assessment on mental health services than on physical health services (AOR = 0.024; 95% CI = 0.259–0.910). The problem of neglecting voices of patients with mental disorders in medical decision-making were consistently reported in previous studies in China (42–47). One major reason is the prevailing concern on patients' insight (48). As a result, family members tend to play a more important role than patients themselves in informed consent and shared decision in China' psychiatric setting (42, 44–47).

### Value and implications of the research

This research has been an important addition to the literature on mental health services both in China and globally. On one hand, our results have demonstrated that mental health services are able to be provided with non-inferior or even better quality than physical health services in the real world. On the other hand, based on the inter-sectoral comparison, our findings would be helpful for informing quality improvement in mental health services.

Our results demonstrated that neglecting patients' voice in medical decision-making was more severe in mental health services. Though some recent studies have reported preference for shared decision-making among patients mental disorders in China (43, 48), how to implement share decision-making in psychiatric setting is still unclear with several challenges (42, 49). Therefore, implementation research to support shared decision-making in routine mental health is needed and the future quality improvement program of mental health services should place more efforts on protecting patients' right of shared decision-making.

### Limitations

The participant recruitment was conducted in one of the best tertiary hospitals in middle south part of China and patients from both departments had a higher socio-economic status than the average population level in China (50). As socio-economic status has a significant relationship with healthcare-seeking behavior (51, 52), participants for the present study are more likely to utilize health services provided by high-quality hospitals, like the survey hospital. Therefore, though 29.9% patients from the PD and 50.0% patients from the ED had inpatient experiences in multiple hospitals, our findings should be cautiously generalized to comparison between mental and physical health services at tertiary level only, rather than the whole system covering services at lower levels. Future comparison between the two types of services should be conducted in an extended scale, covering healthcare providers at different levels in China.

Meanwhile, there was a potential recall bias, as the time frame of the responsiveness performance questionnaire was set to be the past 3 years. However, this was a compromise, in order to maximally capture patients' multiple healthcare encounters and to report quality of inpatient services beyond one hospital.

## Conclusion

Our study reveals that mental and physical health inpatient services provided by China's tertiary hospitals could have similar patient-reported quality, regarding confidentiality of personal information, communication with patients, social support during hospitalization, quality of basic amenities and prompt provision of healthcare. Mental health services could even outperform physical health services on patients' dignity in clinical interaction and patients' choice of healthcare providers. However, the problem of neglecting patients' voice in medical decision-making is more severe in mental health services than physical health services.

## Data availability statement

The raw data supporting the conclusions of this article will be made available by the authors, without undue reservation.

## Ethics statement

The studies involving human participants were reviewed and approved by Institutional Review Board of the Xiangya School of Public Health, Central South University (XYGW-2018-01). The patients/participants provided their written informed consent to participate in this study.

## Author contributions

WZ and SX conceptualized the study. WZ and BL collected the data. WZ, GX, and FO analyzed the data. WZ wrote the manuscript. All authors reviewed the manuscript.
