# Supplementary material for: A comparison of patient-reported quality between inpatient services for mental and physical health: A tertiary-hospital-based survey in China
Source: Front Psychiatry. 2023 Feb 9;14:1090892. doi: 10.3389/fpsyt.2023.1090892 (PMC9949676; doi:10.3389/fpsyt.2023.1090892)
Supplement: Supplementary file 1 [file Table_1.DOCX]

**Appendix 1. Comparison of reporting behaviors on responsiveness between patients from the PD and ED**

| **Domain/item** | **Patients from the PD ^a^**  **n (%)** | **Patients from the ED ^a^**  **n (%)** | **chi-square/df** | **P** |
| --- | --- | --- | --- | --- |
| Domain: Dignity |  |  |  |  |
| Q1. Treating with respect | 20 (12.0) | 13 (10.7) | 0.117/1 | 0.732 |
| Q2. Physical privacy during examination & treatments | 69 (41.6) | 56 (46.7) | 0.736/1 | 0.391 |
| Domain: Confidentiality |  |  |  |  |
| Q3. Talking privately, without overhearing | 12 (7.3) | 7 (5.9) | 0.214/1 | 0.644 |
| Q4. Keeping patient information confidential | 16 (9.8) | 11 (9.2) | 0.021/1 | 0.885 |
| Domain: Communication |  |  |  |  |
| Q5. Communication understandable | 25 (15.2) | 16 (13.3) | 0.205/1 | 0.651 |
| Q6. Enough time for questioning | 45 (27.4) | 48 (40.3) | 5.199/1 | 0.023 |
| Domain: Autonomy |  |  |  |  |
| Q7. Information of alternative treatments or tests | 91 (55.5) | 81 (68.1) | 4.578/1 | 0.032 |
| Q8. Asking user’s opinions when making decisions | 79 (48.2) | 74 (62.2) | 5.454/1 | 0.020 |
| Domain: Choice |  |  |  |  |
| Q9. Choosing a healthcare provider | 28 (17.1) | 26 (21.8) | 1.019/1 | 0.313 |
| Domain: Social support |  |  |  |  |
| Q10. Family/friend visits during hospitalization | 99 (60.4) | 73 (61.3) | 0.028/1 | 0.868 |
| Q11. Contacting with outside during hospitalization | 105 (64.4) | 77 (65.3) | 0.021/1 | 0.885 |
| Domain: Quality of basic amenities |  |  |  |  |
| Q12. Cleanliness of inpatient wards | 14 (8.5) | 4 (3.3) | 3.116/1 | 0.078 |
| Q13. Overall comfortableness of inpatient wards | 10 (6.1) | 9 (7.5) | 0.231/1 | 0.631 |
| Domain: Prompt attention |  |  |  |  |
| Q14. Travelling time to hospital | 8 (4.8) | 10 (8.2) | 1.369/1 | 0.242 |
| Q15. Waiting time of being admitted | 8 (4.8) | 9 (7.4) | 0.837/1 | 0.360 |

^a^ Rating as “good performance”

**Appendix 2. Comparison of samples retained and lost in the post-charge telephone survey**

| **Variables** | **Patients from the PD** | | |  | **Patients from the ED** | | |
| --- | --- | --- | --- | --- | --- | --- | --- |
|  | **Retained (n=168)** | **Lost (n=66)** | **P*** |  | **Retained (n=132)** | **Lost (n=49)** | **P*** |
| Age, mean, median (IQR) | 35.3, 32 (23, 47) | 40.5, 43 (28, 53) | 0.016 |  | 53.3, 55 (43, 65) | 60.1, 63 (54, 67) | 0.007 |
| Male, n (%) | 83 (49.4) | 26 (39.4) | 0.167 |  | 66 (50) | 22 (44.9) | 0.542 |
| Marital status ^a^, n (%) |  |  |  |  |  |  | 0.058 |
| Married | 90 (53.6) | 41 (62.1) | 0.236 |  | 102 (77.3) | 44 (89.8) |  |
| Unmarried | 78 (46.4) | 25 (37.9) |  |  | 30 (22.7) | 5 (10.2) |  |
| Education, n (%) |  |  | 0.107 |  |  |  | 0.592 |
| ≤Primary school | 12 (7.1) | 7 (10.6) |  |  | 42 (31.8) | 16 (32.7) |  |
| Junior high school | 39 (23.2) | 23 (34.8) |  |  | 33 (25.0) | 12 (24.5) |  |
| Senior high school | 49 (29.2) | 11 (16.7) |  |  | 28 (21.2) | 14 (28.6) |  |
| ≥University | 68 (40.5) | 25 (37.9) |  |  | 29 (22.0) | 7 (14.3) |  |
| Employment ^b^, n (%) |  |  | 0.049 |  |  |  | 0.021 |
| Employed | 69 (41.1) | 18 (27.3) |  |  | 46 (35.1) | 8 (17.0) |  |
| Unemployed | 99 (58.9) | 48 (72.7) |  |  | 85 (64.9) | 39 (83.0) |  |
| Years of treatment, mean, median (IQR) | 3.0, 1 (0, 5) | 2.3, 1 (0, 3) | 0.601 |  | 6.8, 2 (0, 11) | 6.9, 4 (0, 11) | 0.487 |
| Hospitalization in multiple hospitals in past 3 years, n (%) | 50 (29.9%) | 21 (31.8) | 0.779 |  | 66 (50.0%) | 27 (55.1) | 0.542 |

IQR interquartile range

*P value by χ2 tests or Mann–Whitney U tests

^a^ Married includes married and cohabited, unmarried includes single, divorced, and widowed

^b^ Employed includes employed and self-employed, unemployed includes unemployed and retired.
